# Morbidity, life style and psychosocial situation in cancer survivors aged 60-69 years: results from The Nord-Trøndelag Health Study (The HUNT-II Study)

**DOI:** 10.1186/1471-2407-11-34

**Published:** 2011-01-26

**Authors:** Ellen K Grov, Sophie D Fosså, Alv A Dahl

**Affiliations:** 1 Department of Clinical Cancer Research, The Norwegian Radium Hospital, Oslo University Hospital, Oslo, Norway; 2 Buskerud University College, Faculty of Health, Drammen, Norway; 3 Faculty Division, The Norwegian Radium Hospital, University of Oslo, Oslo, Norway

## Abstract

**Background:**

Due to considerable health status differences in the elderly population, research limited to narrow age-spans might be an advantage. In this population-based controlled study we compare short-term (<5 years) (STS) and long-term (≥5 years) (LTS) cancer survivors and cancer-free controls aged 60-69 years from two Norwegian health registers; the Health Survey of North-Trøndelag County (HUNT-2 study) and the Cancer Registry of Norway (CRN). We examined possible factors associated with being cancer survivor.

**Methods:**

Among 9,089 individuals aged 60-69 who participated in HUNT-2, 334 had been diagnosed with invasive primary cancer from 1 month to 42 years before HUNT-2 according to CRN and self-report. An overall random sample of controls without cancer five times larger than the sample of cases (N = 1,670) were drawn from the parent cohort.

**Results:**

The cancer sample comprised 128 STS and 206 LTS. For most variables no significant differences were observed between LTS and STS. LTS were significantly more women, and cases with gynaecological cancer, with physical impairment and more thyroid diseases compared to STS. When comparing all the survivors with controls, the survivors showed significantly higher rate of pensioning, decreased self-rated health, more physical impairment and thyroid diseases, daily use of medication and psychotropics and higher level of anxiety and Framingham Risk score. Multivariate logistic regression analysis showed that increasing age, being female, physical impairment and thyroid diseases all were significantly associated with being survivor versus controls.

**Conclusion:**

STS and LTS showed mostly similar situation. Compared to controls, the survivors reported somewhat poorer physical and mental health, but these differences were of doubtful clinical significance.

## Background

In Norway 74% of those who are diagnosed with cancer, are 60 years or older [1], and the life expectancy is 78.2 years in men and 82.7 years in women [2]. Elderly Norwegians who get cancer, therefore often have a considerable lifetime ahead, and studies of their health and psychosocial situation are of substantial interest for prevention of morbidity and possible side effects of treatment. *Cancer survivorship *can be seen as an experience with different phases [3]. The concept may comprehend a stage of living with cancer with or without "treatment" or management [4], or concern patients with cancer "that is controlled with ongoing or periodic treatment" [5]. In most cases five years beyond diagnosis [6] or from end of primary treatment [7] are suggested as the border between short- and long-term survivorship.

Rowland & Bellizzi (2008) [8] highlight the cancer survivors' situation as a melting pot with ingredients of more or less successful coping with bodily, social, mental, existential and economic aspects in life. Consequences of cancer diagnoses and treatment may persist over time with fatigue, digestive problems, sexual dysfunctions, body image changes, comorbidity like cardiovascular disease and osteoporosis, as well as changed attitudes to life as a whole. Several population-based studies of cancer patients have described their psychosocial situation and morbidity, but most of them cover large age intervals. Hewitt et al. reported findings from the American National Health Interview Study [9] comparing cancer patients aged 18 to 75 years to individuals without cancer. Their main findings, and thereby the core challenges in cancer survivors, are poorer health, more functional limitations, higher prevalence of comorbid medical diagnoses, and limitations in performing activities of daily life. However, an age span between 18 and 75 years makes it difficult to specify findings relevant to young, middle-aged or elderly survivors, as their situation and expectations vary considerably with the different phases of life [10]. Blank & Bellizzi [11] studied prostate cancer survivors aged 47 to 88 years and found that increasing age was moderately associated with comorbidity. An optimally designed study includes stratification of some variables in order to create a homogenous sample. In our study we selected cancer survivors in the age span 60-69 for specifications related this age category, which in Norway represent the end of active work life for the majority of people. Alfano et al. (2007) [12] report on a sample of cancer survivors aged 29 to over 70 years without age stratification of the analyses. This sample will certainly allow for reflections about the influence of age related concerns when studying morbidity, psychosocial aspects and lifestyle.

Deimling et al. [13] studied older adult survivors (mean age 72.3 years, range <60 - >75 years) reporting that elderly cancer survivors were vulnerable for functional difficulties, and to comorbid health conditions, where pain was the most common symptom attributed to cancer or cancer treatment. Sweeney et al. [14] focused on elderly female cancer survivors, 57% of them older than 68 years (mean age 72 years, range 66-82 years), who carry a risk of confounding of age-related morbidity with the effects of cancer and its treatment. Several functional limitations were significantly more frequently reported among elderly female cancer survivors compared to controls without cancer. In a study among elderly cancer survivors, ≥65 years, Grov et al. [15] showed significant associations between somatic comorbidity, lifestyle, and somatic symptoms.

In order to prevent morbidity and long-term effects after cancer and its treatment, health care personnel should be aware of cancer survivors' vulnerability for health problems, unfavorable life style, and psychosocial challenges. These are factors that we hypothesize to be associated with the survivors' age and time since diagnosis.

We hold the view that morbidity studies of cancer survivors should be restricted to defined age spans. Therefore, this study focused on cancer survivors aged between 60 and 69 years. This decade includes regular retirement (from 62 to 69 years of age in Norway), and increasing risk of somatic morbidity and symptomatology. In the decade from 60 to 69 years, the rate of cognitive impairment is low, and self-report is therefore a reliable mode of data collection. Lifestyle changes are still possible with the aim to decrease the risk for cardiovascular comorbidity and long-term health effects after cancer treatment [16,17].

An ideal study would have a longitudinal design with follow-up for the selected groups of cancer survivors (60-69 years) to be continued for ten years (to the survivors reach the age of 70-79). The study should have no dropouts, and the treatment given to all cancer patients should be well documented, and all relevant variables for the study of morbidity, lifestyle and psychosocial situation must be covered. Norway has special opportunities in research on cancer survivors due to the unique person number allowing for linkage between high quality health-related registries. In this study, persons aged 60 to 69 years participating in the Health Survey of Nord-Trøndelag County 1995-97 (HUNT-2) were linked to the Cancer Registry of Norway (CRN).

Since the dataset from the HUNT-study covers many relevant variables about morbidity, psychosocial situation and lifestyle, we considered that our study could give valid information regarding several aspects concerning the situation of cancer survivors. All these variables are relevant for planning health care service for cancer survivors. Compared to other studies in the field, our study had the following advantages: internationally accepted schedules like the HADS, the Rosenberg instrument, and the Framingham Risk Index, in addition to a variety of somatic symptoms included by the gold standard of self-report, and a valuable link to cancer specific register data by the Cancer Registry of Norway (CRN) which contributes with data on quite reliable cancer diagnoses.

The aim of the study was to examine morbidity, lifestyle and psychosocial situation in a sample of patients with invasive cancer compared to a random sample of controls without cancer. 1) We first compared cancer patients with primary diagnosis <5 years before HUNT-2 (Short Term Survivors, STS) to those who had their diagnosis ≥5 years before HUNT-2 (Long Term Survivors, LTS). 2) We then compared the cancer survivors (SURV) with controls without cancer (controls). Our hypotheses were for 1) that no significant differences between short- and long-term survivorship would be demonstrated, and for 2) that SURV would show more morbidity, and similar lifestyle and psychological situation as controls.

## Methods

### Sampling

The second Health Survey of Nord-Trøndelag County (HUNT-2) invited all inhabitants of the County aged 20 years and above to a health survey. The survey was done locally through the 24 municipalities of the County between August 1, 1995 and June 30, 1997. The personal invitation provided time and place for a simple physical examination, non-fasting blood sampling, and also included a questionnaire (Form 1), to be filled in and delivered at the examination. At the examination a second questionnaire (Form 2) was handed out to be returned by prepaid mail. Details of the HUNT-2 study are given elsewhere [18], (http://www.hunt.ntnu.no). Within the 60-69 years age group 10,611 individuals were invited to HUNT-2, and 9,089 (86%) participated.

Report of all cancer cases occurring in Norway to the CRN has been mandatory by law since 1953, and the CRN is considered as a quite complete and reliable registry concerning cancer localization and invasiveness. Based on the person numbers an authorized linkage between HUNT-2 and the CRN, identified 428 participants (5%) with at least one diagnosis of invasive cancer diagnosed >1 month before their HUNT-2 examination. Basal cell and squamous cell carcinomas (skin cancer) were excluded due to its superficial character not incorporated in our way of defining 'invasive cancer', which means more aggressive invasion into the tissue. From the 428 we excluded 71 participants found in the CRN but who did not self-report cancer, and 23 participants who had not filled in Form 2. This left us with a sample of 334 cancer patients (cases), among them 128 (38%) had been diagnosed with cancer <5 years before HUNT-2 (STS group) and 206 (62%) ≥5 years before HUNT-2 (LTS group).

Among the 8,661 participants in the parent cohort not registered in the CRN, we excluded 56 who reported cancer but did not have a record in the Registry, and 609 participants who did not fill in Form 2. From the rest of 7,996 participants in the parent sample, we drew an overall random sample of five controls for each case, and these 1,670 participants represented the control group (NORM).

### Variables

#### Demographic variables

*Civil status *was dichotomized into those married and those single, separated, divorced or widowed. *Level of basic education *was dichotomized into < 10 years and ≥10 years. The *work situation *was divided into those being in paid work or independent business versus those on age or disability pension. *Economic problems last year *were defined as reported problems paying bills during that time. *Social network *was assessed by having enough friends or not, and *social activities *were defined as being active in social clubs ≥1 time/month or not.

#### Lifestyle variables

*Daily smoker *was registered for persons who reported daily consumption of cigarettes. *Body Mass Index (BMI) *was calculated. The level of *physical activity *was divided into "minimal" and "moderate or more" according to Thorsen et al. [19]. The "minimally active" category represents not sweaty/breathless activity ≥1 hour per week, and either no or <1 hour per week of higher-level activity (sweaty/breathless). The "moderate or more" category is defined by higher-level activity ≥1 hour per week. *The Framingham Risk Index *score was calculated for males and females according to algorithms of the Adult Treatment Panel [20], which includes weighted assessments of cholesterol, high density cholesterol, smoking and blood pressure, and indicates to what degree a person is at risk for developing coronary heart disease or sudden cardiac death within 10 years.

#### Somatic morbidity variables

*Impairment *was defined as caused by chronic disease, injury or somatic or mental morbidity leading to reduced activity of daily living, and divided into mainly *physical *or *mental *impairment. *Self-rated health *was rated to be "good" (very good/good) or "poor" (poor/very poor). *Somatic diseases *were asked for by the question: "Has a doctor ever said that you had...?": myocardial infarction, angina pectoris, stroke, diabetes, thyroid disease (either hyperthyroidism, hypothyroidism, goiter or other thyroid diseases), osteoporosis, arthritis/arthroses (rheumatoid arthritis, arthrosis or ankylosing spondylitis), and musculo-skeletal diseases (including fibromyalgia). A diagnosis of either myocardial infarction, angina pectoris, or stroke, qualified for a diagnosis of cardiovascular disease. *Comorbid disease(s) *were present if the patients had ≥1 of these diseases. *Somatic symptoms *covered *gastrointestinal *ones (nausea, heartburn, diarrhea, or constipation) if they had caused "much bother last year", and *headache *defined as attacks during the last year. *Muscular pain and stiffness affecting activities of daily living *last month was rated as present or absent.

*Regular use of medication last year *concerned daily use of any medication, and regular use of antihypertensives, analgesics, and psychotropics (hypnotics, anxiolytics, and/or antidepressants) were registered. Daily use refers to daily consumption during the last 12 months.

#### Mental distress variables

*Anxiety and depression *were self-rated with The Hospital Anxiety and Depression Scale (*HADS). *The psychometric properties of the scale had been tested in HUNT-2 [21,22]. Scores on the anxiety and depression sub-scales ranged from 0 to 21, respectively, and higher scores mean increased symptom load. HADS-defined caseness of anxiety and depression were identified by a cut-off ≥8 on the subscale scores [21]. The correlation between the anxiety and depression subscales were Spearman's r = 0.62. Internal consistency by Cronbach's coefficient α was 0.85 for the anxiety-subscale, and 0.78 for depression.

*Rosenberg self-esteem score *was assessed with four of the 10 items introduced by Rosenberg [23]. These items were self-rated on four-point Likert scales ranging from "strongly agree" to "strongly disagree" [24], and the score ranged from zero to 12, with higher scores meaning better self-esteem. Internal consistency measured by Cronbach's coefficient α was 0.69.

*Sleeping problems *were present if they regularly occurred for one or more nights a week. *Alcohol problems *were present in respondents who scored positively on one or more of the four CAGE items [25,26].

### Data from the Cancer Registry (CRN)

For all cancer patients the following data were collected from the CNR: *1) date of first cancer and the interval *to their HUNT-2 participation; 2) *localization of cancer *were categorized into six groups according to organ systems, where skin cancers were excluded.

### Statistical analysis

Data were analyzed by SPSS-PC, version 15.0. Continuous variables were examined with t-tests, and categorical variables with χ^**2**^-tests. Non-parametric tests were applied in case of skewed distributions. Statistically significant group differences were examined for clinical significance by means of effect sizes (ESs) [27]. Clinical significance is the conclusion that a group difference has an effect of practical meaning to patients and health care providers. Even though a group difference is found to be statistically significant, this difference may not be clinically significant. For continuous variables we used Cohen's coefficient d, and for 2 × 2 contingency tables the differences between arcsine transformed proportions and ES values ≥0.40 were considered as clinically significant based on the recommendations of Cohen [28-30].

Internal consistencies of scales and subscales were examined by Cronbach's coefficient α. A standard (unconditional) multivariate logistic regression analysis examined the associations between the dependent variable (cancer survivors or controls), and the selected independent variables showing significant associations in the bivariate analysis. Strength of association was expressed as odds ratio (OR) with 95% confidence interval (95% CI). The significance level was set at p < 0.05, and all tests were two-tailed.

### Ethics

The HUNT-2 study was approved by The Norwegian Data Inspectorate and by The Regional Committee for Medical Research Ethics, Health Mid-Norway. All participants in HUNT-2 gave written informed consent.

## Results

### Characteristics of the cancer survivors

No significant age difference was found between STS and LTS. The mean time from diagnosis of cancer to the HUNT-2 examination was 2.3 years (median 2.1) among STS and mean 13.8 (median 12.6) among LTS, which was to be expected considering the definitions of the STS and LTS. The time interval between diagnosis and HUNT-2 was significant longer in female cancer survivors, and they had lived a mean of 12 years (median 8.5) since their primary diagnosis. For male survivors the comparable mean was 7.5 years (median 5.0). Analyses on 5-year survival show that of the 334 participants in this study 78 have died of cancer, where 38 of them have survived less than 5 years (data until 2005).

The most common localizations of cancer were breast, gastrointestinal tract, and the group of other organ systems. STS contained significantly less gynaecological cancer (ES = 0.41) and more prostate cancer (ES = 0.62) than LTS, and both these findings showed clinical significance.

### Comparisons of STS and LTS

There were significantly more females among LTS than STS (ES = 0.33), and therefore all analyses comprising morbidity and lifestyle variables are adjusted for sex. Concerning other socio-demographic, work, and social activity variables no significant differences were observed between LTS and STS (Table 1).

**Table 1:** Demographic and clinical characteristics of short- and long-term cancer survivors

Variables	Short-term survivors (N = 128)	Long-term survivors (N = 206)	p-value	Effect Size
Age, mean (SD)	65.3 (2.9)	65.0 (3.0)	0.28	

*Time since diagnosis (years)*				
Mean (SD)	2.3 (1.4)	13.8 (7.2)	<0.001	2.00
Median	2.1	12.6		
Range	0.1 - 5.0	5.1 - 42.2		

*Localization of cancer, N (%)*				
Respiratory tract	5 (4)	11 (5)	0.79	
Gastrointestinal tract	28 (22)	32 (15)	0.14	
Breast	33 (26)	57 (27)	0.80	
Gynecological	13 (10)	52 (25)	0.001	0.41
Prostate	24 (19)	5 (2)	<0.001	0.62
Other organ systems	25 (20)	49 (23)	0.41	

*Sex, N (%)*			0.002	0.33
Male	57 (44)	58 (28)		
Female	71 (56)	148 (72)		

*Civil status, N (%) *			0.95*	
Married	95 (74)	147 (71)		
Single, separated, divorced, widowed	33 (26)	59 (29)		

*Level of basic education, N (%)*			0.66*	
<10 years	76 (63)	130 (68)		
≥10 years	45 (37)	61 (32)		

*Work status, N (%)*				
Paid work, independent business	37 (29)	51 (25)	0.71*	
Disability or age pension	89 (70)	144 (70)	0.95*	

*Economic problems last year, N (%)*	14 (11)	22 (11)	1.00*	

*Have enough friends, N (%)*	106 (82)	161 (78)	0.06*	

*Active in social clubs ≥1 time/month, N (%)*	47 (40)	94 (51)	0.11*	

*Adjusted for sex

As to somatic variables LTS showed significantly more thyroid diseases (ES = 0.36) and more physical impairment (ES = 0.24) than STS, but no other somatic variables showed significant group differences (Table 2). Neither was any significant differences observed concerning daily use of any medication or regular use of analgesics, psychotropics or antihypertensive medication.

**Table 2:** Somatic and mental morbidity of short- and long-term cancer survivors

Variables	Short-term survivors (N = 128)	Long-term survivors (N = 206)	p-value*	Effect Size
*Self-rated health*			0.62	
Good health	59 (47)	92 (45)		
Poor health	66 (53)	112 (55)		

*Functional impairment*				
Physical impairment	16 (13)	45 (22)	0.04	0.24
Mental impairment	8 (6)	15 (7)	0.89	

*Somatic diseases*				
Infarction, angina or stroke	18 (14)	26 (13)	0.96	
Diabetes	5 (4)	9 (4)	0.82	
Thyroid diseases	9 (7)	39 (19)	0.01	0.36
Osteoporosis	3 (2)	14 (7)	0.16	
Arthritis, arthrosis	31 (24)	49 (24)	0.62	
Musculo-skeletal diseases	18 (14)	36 (18)	0.45	
≥1 comorbid disease(s)	57 (45)	112 (54)	0.12	

*Significant somatic symptoms*				
Muscular pain and stiffness affect-				
ting daily activities last month	18 (14)	48 (23)	0.05	
Gastrointestinal symptoms last year	11 (9)	16 (8)	0.82	
Headache last year	24 (19)	52 (25)	0.35	

*Regular use of medication last year*				
Daily use of any medication	76 (59)	114 (53)	0.55	
Analgesics	21 (16)	31 (15)	0.83	
Psychotropics	25 (19)	43 (21)	0.99	
Antihypertensives	25 (20)	50 (24)	0.33	

*Mental variables*				
HADS-Anxiety, mean (SD)^a^	4.8 (4.1)	4.8 (3.7)	0.78	
HADS-Depression, mean (SD) ^a^	4.1 (3.3)	4.4 (3.2)	0.56	
Self-esteem score, mean (SD)	7.9 (2.1)	7.7 (2.2)	0.54	
Sleeping problems, N (%)	30 (25)	59 (32)	0.33	
Alcohol problems, N (%)	8 (6)	17 (8)	0.15	

*Lifestyle variables*				
BMI, mean (SD)	27.6 (3.8)	27.6 (4.8)	0.70	
*Physical activity, N (%)*				
Minimal	40 (31)	80 (39)	0.63	
Moderate or more	88 (69)	126 (61)	0.48	
*Daily smoker*	26 (20)	55 (27)	0.38	
*Framingham risk score*				
Framingham sum score, mean (SD)	16.1 (2.8)	16.6 (3.0)	0.15	
Framingham risk >20%, N (%)	69 (54)	123 (60)	0.48	

*Adjusted for sex ^a ^Non-parametric test

No significant differences concerning mental morbidity or lifestyle variables were observed between LTS and STS (Table 2).

### Comparisons between cancer survivors and controls

Since only two variables among socio-demographic, somatic, mental and lifestyle ones showed significant differences (thyroid diseases and physical impairment), we found it allowable to analyze LTS and STS taken together as survivors versus controls.

The survivors was significantly older than the controls (ES = 0.24), and there were significantly more females among survivors (ES = 0.26). In the analyses of other variables we adjusted for age and sex. Only two significant differences among socio-demographic were observed namely that survivors had a higher proportion on pensions (ES = 0.25) and a lower proportion with economic problems (ES = 0.17) than the controls (Table 3).

**Table 3:** Demographic, work, social activity and health care consumption s of cancer survivors and controls

Variables	Survivors (N = 334)	Controls (N = 1,670)	p-value	Effect size
Age at survey, mean (SD)	65.1 (2.9)	64.4 (2.9)	<0.001	0.24

	*N (%)*	*N (%)*		

*Sex*			<0.001	0.26
Males	115 (34)	792 (47)		
Females	219 (66)	878 (53)		

*Civil status*			<0.43*	
Married	242 (73)	1,280 (77)		
Single, separated, divorced, widow(er)	92 (27)	387 (23)		

*Level of basic education*			0.77*	
<10 years	206 (66)	953 (62)		
≥10 years	106 (34)	583 (38)		

*Work situation*				
Paid work, independent business	88 (26)	593 (36)	0.30*	
Disability or age pension	233 (70)	961 (58)	0.006*	0.25

*Economic problems last year*	36 (12)	288 (18)	0.02*	0.17

*Have enough friends*	267 (87)	1,396 (87)	0.75*	

*Active in social clubs ≥1 time/month*	141 (46)	761 (48)	0.33*	

* Adjusted for age and sex

Significantly more survivors than controls reported poor self-rated health (ES = 0.26), physical impairment (ES = 0.26) and thyroid diseases (ES = 0.23) compared to controls (Table 4), but none of these differences showed clinical significance (Table 4).

**Table 4:** Somatic and mental morbidity of cancer survivors and controls

Variables	Survivors (N = 334)	Controls (N = 1,670)	p-value	Effect size
*Self-rated health*			<0.001*	0.26
Good health	151 (46)	971 (59)		
Poor health	178 (54)	687 (41)		

*Functional impairment*				
Physical impairment	76 (23)	230 (13)	<0.001*	0.26
Mental impairment	14 (4)	62 (4)	0.62*	

*Somatic diseases*				
Infarction, angina or stroke	44 (13)	223 (13)	0.85*	
Diabetes	14 (4)	73 (4)	0.83*	
Thyroid diseases	48 (14)	121 (7)	0.001*	0.23
Osteoporosis	17 (5)	65 (4)	0.84*	
Arthritis, arthrosis	80 (24)	408 (24)	0.34*	
Musculo-skeletal diseases	54 (16)	207 (12)	0.07*	
≥1 comorbid disease(s)	169 (51)	809 (48)	0.99*	

*Significant somatic symptoms*				
Muscular pain and stiffness affecting				
activities of daily living last month	66 (20)	322 (19)	0.70*	
Gastrointestinal symptoms last year	36 (11)	165 (10)	0.82*	
Headache last year	76 (23)	428 (26)	0.19*	

*Regular use of medication last year*				
Daily use of any medication	190 (57)	822 (49)	0.01*	0.16
Analgesics	45 (14)	179 (11)	0.15*	
Psychotropics	56 (17)	187 (11)	0.02*	0.17
Antihypertensives	75 (23)	361 (22)	0.98*	

*Mental variables*				
HADS-Anxiety, mean (SD)^a^	4.8 (3.8)	4.0 (3.4)	0.001*	0.20
HADS-Anxiety caseness, N (%)	77 (25)	208 (13)	<0.001*	0.31
HADS-Depression, mean (SD) ^a^	4.2 (3.3)	4.1 (3.2)	0.41*	
HADS-Depression caseness, N (%)	47 (15)	230 (14)	0.73*	
Self-esteem score, mean (SD)	5.6 (1.9)	5.7 (1.6)	0.39*	
Sleeping problems, N (%)	89 (29)	385 (24)	0.35*	
Alcohol problems, N (%)	25 (8)	147 (8)	0.57*	

*Lifestyle variables*				
BMI, mean (SD)	27.6 (4.5)	27.4 (4.2)	0.84*	
*Physical activity, N (%)*			0.42*	
Minimal	86 (31)	475 (34)		
Moderate or more	188 (69)	930 (66)		
*Daily smoker*	64 (23)	308 (22)	0.49*	
*Framingham risk score*				
Framingham sum score, mean (SD)	16.4 (2.9)	16.0 (2.9)	0.01	0.14
Framingham risk >20%, N (%)	198 (58)	867 (52)	0.12	

* Adjusted for age and sex a Non-parametric test

The proportions of individuals that used daily medication (ES = 0.16), analgesics, or psychotropics (ES = 0.17) were all significantly higher among survivors compared to controls. However these differences did not reach clinical significance.

Survivors showed significantly higher level of anxiety (ES = 0.20) and more HADS-defined cases of anxiety (ES = 0.31) than controls. The other mental variables did not show any significant differences between the groups (Table 4).

No significant differences between the groups were observed for any of the lifestyle variables, except for The Framingham Risk Index mean score which was significantly higher in survivors compared to controls (ES = 0.14). However, the proportions with a risk >20% did not differ significantly between the groups.

The standard multivariate logistic regression analysis with 'survivors versus controls' as dependent variable showed that increasing age, female sex, lack of economic problems, physical impairment, and presence of thyroid diseases were significantly associated with the dependent variable (Table 5).

**Table 5:** Multivariate logistic regression analysis with cancer survivors (N = 334) versus controls (N = 1,670) as dependent variable

Independent variables	OR	95% CI	*P*
Age	1.09	1.03 - 1.15	0.003
Female (male = reference)	1.57	1.12 - 2.20	0.009
Pensioned (not pensioned = reference)	1.28	0.92 - 1.37	0.14
Economic problems last year	0.54	0.36 - 0.80	0.002
Poor self-rated health (good = reference)	1.27	0.95 - 1.71	0.11
Physical impairment present	1.66	1.17 - 2.37	0.005
Thyroid diseases	2.05	1.39 - 3.03	<0.001
Daily use of psychotropics	1.29	0.89 - 1.88	0.18
HADS Anxiety	1.03	0.99 - 1.07	0.14
Framingham risk score	0.95	0.89 - 1.00	0.07

## Discussion

This study of cancer survivors aged 60-69 years and a random sample of controls had two main findings: 1) Among LTS there were significantly more females, and more cases with gynecological cancer, physical impairment, thyroid diseases, but fewer cases with prostate cancer than among STS. Only the differences concerning cancer types reached clinical significance, however they represent 94/334 patients (28%), which leaves limited reason to believe, that types of treatment differed systematically or significantly between the groups of survivors. 2) A considerable number of significant differences were observed between cancer survivors and controls. The survivors were significantly older and had a higher proportion of females than controls. Among survivors a significant higher proportion were pensioned, had poorer self-rated health, physical impairment, thyroid diseases, daily use of medication and psychotropics, and HADS-defined anxiety disorders than controls. The mean scores on anxiety and Framingham Risk Index were also significantly higher among survivors than controls. None of these differences showed clinical significance, however. We conclude that survivors in the age group of 60-69 years are worse of in several respects compared to controls. However, the clinical consequences of these differences are in need of further investigation. In multivariate analysis having thyroid diseases was significantly and positively associated with being survivor, and such diseases were significantly more common among LTS than STS, so investigations of the thyroid and follow-up of such diseases seems worthwhile in the group of survivors.

Somewhat poorer self-rated health and more physical impairment in survivors than among controls are in line with the findings from the study by Hewitt et al. [9], where especially the subgroup ≥65 years with a history of cancer reported significantly poorer health and more disability than age-matched controls without cancer. Our results support the study by Yabroff et al. [31] in which cancer survivors reported significantly poorer health compared to matched controls. Yabroff et al. also concluded with higher rate of pensioning and less work ability in survivors compared to controls. However, the comparison with that study might be biased due to their large age span (<20 - ≥70 years). The problem regarding comparisons with studies using long age spans is also relevant in relation to other studies such as Hewitt et al. [9], Deimling et al. [13], and Blank & Bellizzi [11].

Except for thyroid diseases, survivors did not have more somatic diseases or bothering somatic symptoms than controls. The thyroid findings might be related to the high proportion of breast cancer patients in the study since a clear association with thyroid diseases has been shown in such patients [32]. In multivariate analysis having thyroid diseases was significantly and positively associated with being survivor, and such diseases were significantly more common among LTS than STS, so investigations of the thyroid and follow-up of such diseases seem worthwhile in the group of survivors. Physical impairment was also significantly associated with survivor status in the multivariate analysis, but since we have no further details on this variable, evaluation and interventions cannot be suggested by us.

No significant differences were found between survivors and controls for cardiovascular diseases or antihypertensive medication. We had expected such difference, or at least significant differences between the groups for Framingham Risk Index. The latter was found for the mean scores, but the ES = 0.14 indicated doubtful clinical relevance, particularly since no significant difference was found for the high risk (>20%) cases. Previous studies have reported increased heart-related comorbidity in cancer survivors, but differences in sampling can explain the divergent findings [13,16,17,33].

We observed no significant differences between survivors and controls on variables that are relevant for lifestyle interventions. The group in need of such interventions must therefore be defined by other variables than survivor status.

As observed in the study of Mehnert & Koch [34], our findings also support the lower level of self-rated health in cancer survivors compared to controls. However, Mehnert & Koch found a significantly higher level of depression in elderly breast cancer survivors compared to controls, while that was not the case in our study (mean 4.4, SD 3.5, and mean 4.1, SD 3.3, p = 0.26 for female survivors and controls, respectively). In contrast, we observed a higher level of anxiety in survivors compared to controls, however not to clinically significant degree. Since the HADS anxiety and depression subscales are highly correlated, the two studies have in common a higher level of mental distress in survivors compared to controls.

To our knowledge there are hardly any studies that compare LTS and STS on the variables examined in this study. Our hypothesis of significant differences between the groups was hardly confirmed. However, the hypothesis of more morbidity among survivors compared to controls was confirmed. Health care personnel should be aware of health problems in cancer survivors by highlighting assessments of their physical and mental health. Poor physical health and mental distress might have implications for the way cancer survivors cope with e.g. performance of activity of daily living, their use of medication, and their social capability.

The few significant differences observed could be due to chance since we did a considerable number of comparisons. If we apply Bonferroni's correction for multiple comparisons we get a p-value of 0.002 (0.05/32), and no significant differences emerge. Few significant differences between LTS and STS could reflect that survivorship is a relatively stable state, but not necessarily without problems. As with other chronic diseases cancer survivors have to cope with challenges coming up now and then, and our findings indicate that cancer survivors find ways to deal with consequences of cancer treatment and side effects during the survivorship trajectory. We find the lack of difference between STS and LTS in a mixed sample of cancer survivors of considerable interest, and this result should be focus for more studies.

The strength of this study is the data collection combining data from a large health survey with a high quality cancer registry. Due to this combination, we also had opportunity to draw at random a control group from the same parent population, which is an advantage for comparison. Population-based studies like HUNT-2 provide the opportunity to study a short age span of cancer survivors with a sufficient sample size. The use of internationally accepted schedules like the HADS, the Rosenberg instrument, and the Framingham Risk Index is an additional strength.

On the other hand, some limitations have to be considered when interpreting the results from this study. One is the large time of survivorship studied ranging from 0.1 - 42.2 years. Fourteen per cent of those aged 60-69 years did not participate in HUNT-2. Among the 1,522 non-responders in the age group 60-69, 225 (14%) had invasive cancer, while in the participant group (9,089) 334 (4%) had invasive cancer. These findings represent a source of systematic bias, and we suggest that one explanation is that they did not want to participate in the survey due to health problems. Therefore we cannot exclude a selection bias concerning the cancer survivors included compared to the survivors in the population. Additionally, more somatic symptoms might be present for the respondents, but they were not available for assessment in HUNT-2.

Our study had a cross sectional design which gives a snapshot of the cancer survivors' situation without opportunities to identify causal connection. We do not have any information concerning the non-responders to HUNT-2. The variables selected for the HUNT-study did not cover all variables relevant for the study of cancer survivors (e.g. pain was not covered). The Cancer Registry of Norway (CRN) does not have valid data on cancer treatment, which would have been highly relevant information. The survey was sampled some years ago. However, we cannot see that this delay influences the aims and results of this study. The cases have been divided into two subgroups under the same conditions related treatment and follow-up. Even if we take into account that new treatment modalities have emerged from the sampling of this study, the pattern of problems and challenges for short- and long-term survivors highlighted in our study still seem appropriate.

## Conclusion

In this controlled population-based study of cancer survivors age 60-69 years, we observed few significant differences between LTS and STS. In accordance with other studies of elderly cancer survivors, we found significant differences between survivors and controls drawn at random within psychosocial, morbidity and lifestyle variables. However, none of these differences reached clinical significance. Anyway, health care personnel are recommended to identify individuals with health problems like thyroid disease among the cancer survivors, and to assess their physical and mental health in order to identify physical diseases and mental distress.

## Conflict of interest statement

The authors declare that they have no competing interests.

## Authors' contributions

EKG, SDF & AAD were responsible for the study conception and design. EKG, SDF & AAD performed the data analysis. EKG, SDF & AAD drafted the manuscript. EKG, SDF & AAD made critical revisions to the paper for important intellectual content. EKG & AAD provided statistical expertise, and EKG obtained funding. AAD & SDF supervised the study. All authors read and approved the final manuscript.

## Pre-publication history

The pre-publication history for this paper can be accessed here:

http://www.biomedcentral.com/1471-2407/11/34/prepub
